# Radiolucent Skull Clamps for Intra-Operative Imaging: A Technical Note

**DOI:** 10.7759/cureus.1020

**Published:** 2017-02-10

**Authors:** Neil Haranhalli, Hugo Zeberg, Patrick Lasala, David Altschul

**Affiliations:** 1 Department of Neurological Surgery, Montefiore Medical Center; 2 Neurological Surgery, Karolinska Institutet

**Keywords:** intraoperative imaging, skull clamp, radiolucent, metal artifact, cone beam ct

## Abstract

Conventionally, surgery and procedural-based radiology are performed on different premises. With advances in imaging technology, the operating room is rapidly being transformed into an intraoperative imaging suite. Diagnostic imaging in conjunction with surgery has great utility and by all accounts has great future potential. During the last decade, cone beam computed tomography (CT) scanners have been introduced and have made intraoperative imaging more feasible because these scanners can be made less bulky. The current usefulness of intraoperative magnetic resonance imaging (MRI) or CT for neurosurgery, however, is impaired by the lack of completely radiolucent skull clamps, causing image artifacts. Metal artifacts are particularly problematic, given that they lead to a considerably higher image quality degradation factor for cone beam CT scanners than for conventional CT scanners. Here, we describe our experience with near-radiolucent skull clamps and their associated problems and discuss future improvements to facilitate high-quality image guidance in the field of neurosurgery.

## Introduction

One of the most commonly used skull clamps today, a three-pin skull clamp that rigidly affixes a patient's head to the operating table was designed by Frank H. Mayfield 40 years ago. The Mayfield skull clamp has proven to be flexible, allowing stable fixation and good accessibility in the supine, prone and oblique positions and it has remained largely unchanged since its creation. The skull clamp and all its parts are usually fabricated from stainless steel. Given the radiopaque nature of steel, such clamps cause X-ray artifacts if the clamps are interposed between the X-ray source and the equipment used for X-ray detection. However, until the introduction of intraoperative imaging, this issue has not been a problem.

Generally, an artifact is any systematic disagreement between a scan and the true attenuation of an object, an artifact can seriously degrade the quality and usefulness of constructed images. The types of artifacts that can occur for X-ray computer tomography can be divided into physics-based, patient-based, scanner-based and helical and multisection CT artifacts [1]. The study mainly focuses on physics-based artifacts and to some extent patient-based artifacts. The streaks observed for metal artifacts are caused by beam hardening, scatter motion, and under-sampling [2-3]. These effects are partially due to high metal attenuation and the high atomic number of the metals used. CT scans of posterior cranial fossa are also problematic, however, stainless steel has a Hounsfield unit of approximately 12,000 [4] compared with approximately 1,000 for dense bone [5]. Sharp thin alternating streaks may be due to motion and under-sampling [2]. Moreover, due to the data handling of beam hardening and scatter, there will be bright streaks in other directions [2]. Streak artifacts particularly occur between two high-attenuation objects such as metal or bone.

In cone-beam CT scanners, X-ray beams form a conical geometry; whereas, in traditional CT scanners, the beam is restricted to a 2D slice. Advantages of this type of scanner include the fact that they can often provide data acquisition in one rotation and can be made less bulky owing to the compact size of the flat panel detector [6]. Because of the more complex geometry of the beam, it is more difficult to compensate for metal artifacts, but novel software techniques are being developed [7]. Conventional CT scanners employ a number of strategies to reduce scatter that cannot be used for a cone-beam CT [6]. Additionally, because of the larger flat-panel detector used, scatter radiation is a larger problem for cone-beam CT [8].

For intraoperative imaging, the artifacts described above present a major problem for surgeons, in reducing the medical usefulness of developed X-ray pictures. To overcome these problems skull clamps have been constructed with radiolucent material; however, they are not 100% radiolucent as we demonstrate in this article. In the description of an improved radiolucent Mayfield skull clamp (Mayfield Clinic and Spine Institute, Cincinnati, Ohio) (U.S. Patent 5276927), it is stated that although it is desirable to eliminate the use of metal, minor structural elements, (e.g. springs and pins) not directly in the path of X-rays may be made of metal [9]. In addition to three-pin skull clamp devices, the Sugita frame, a four-pin skull clamp device has also been developed in a radiolucent version, which is marketed for intraoperative angiography using X-rays [10].

To employ the full potential of portable CT machines in neurosurgical operating rooms, 360 degrees of radiolucency is needed to contrast C-arm, X-ray, or digital subtraction X-ray guided interventions in which computer-assisted tomography is not typically used. In this technical note, we describe our experience with four types of radiolucent skull clamps and the portable BodyTom scanner (NeuroLogica Corporation, Danvers, Massachusetts). Informed consent was obtained from the patient for this study.

## Technical report

Radiolucent skull clamps were obtained from Pro Med instruments (PMI, Freiburg, Germany), the Doro skull clamp (Integra LifeSciences Corporation, Plainsboro, New Jersey, U.S.A), the Mayfield radiolucent skull clamp and the Sugita Carbon fiber skull clamp (Mizuho America, Union City, CA). Skull clamps were assessed for relative radiolucency in both an operating room and at a department of radiology using phantoms. Radiolucent pins for all but the Sugita frame were obtained from Integra (Plainsboro, New Jersey, United States). All four clamps were scanned using conventional CT. One of the Mayfield frames was more closely studied using the BodyTom scanner. 

Near-radiolucent skull clamps examination of water phantoms upon conventional CT

**Figure 1:**
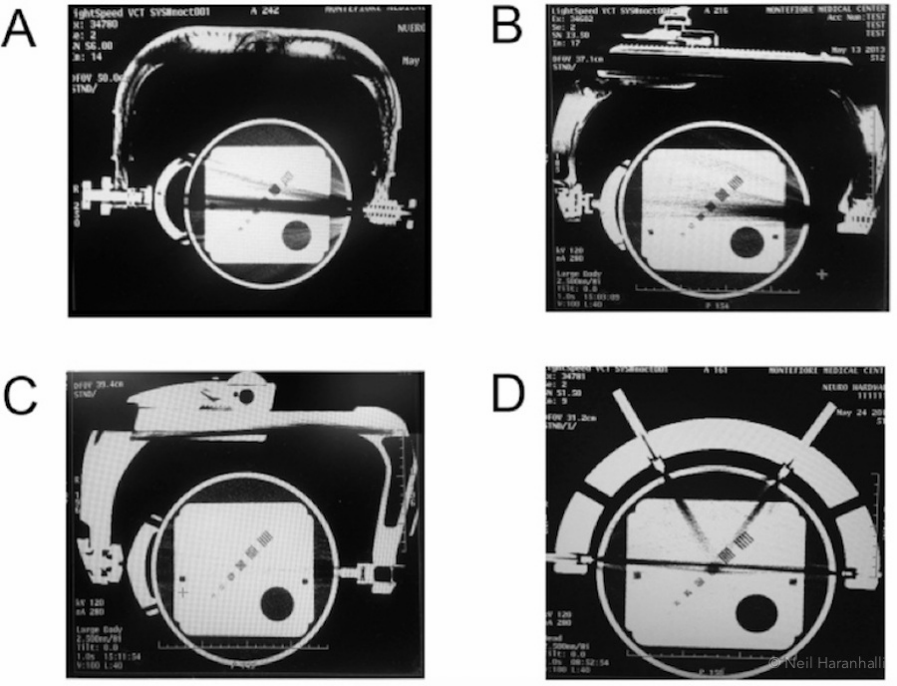
Four near-radiolucent frames in conventional CT Upper row (A and B), two Mayfield skull clamps (C). A radiolucent Doro skull clamp (D). A Sugita carbon fiber head frame. Note the dark streaks along the line of greatest attenuation originating from the metal objects adjacent to the pins

Figure.1 depicts the results of four near-radiolucent skull clamps. Both of the Mayfield clamps and the Doro clamp, although to a lesser extent, (Fig. 1A-C) suffer from metal artifacts due to a spring in the one pinned arm. The Sugita frame (Fig. 1D) has no spring, but pins connected to the rest of the head frame are not radiolucent and show artifacts along the lines from the pins. As observed in Fig. 1, the artifacts are dominated by dark lines (originating from beam hardening and scatter as discussed above). The intensity of the X-ray was set to 120 kV.

Examination of a near-radiolucent skull clamp using the BodyTom scanner: A case report

A 61-year-old female presented with confusion, visual field deficits, and a headache. MRI revealed a left parieto-occipital mass with significant vasogenic edema and smaller enhancing satellite lesions along the midline. There was involvement of the left splenium of the corpus callosum. Smaller foci of enhancement were observed within the left lobe, pons and left subcortical precentral gyrus. A differential diagnosis included a primary and secondary neoplasm.

**Figure 2:**
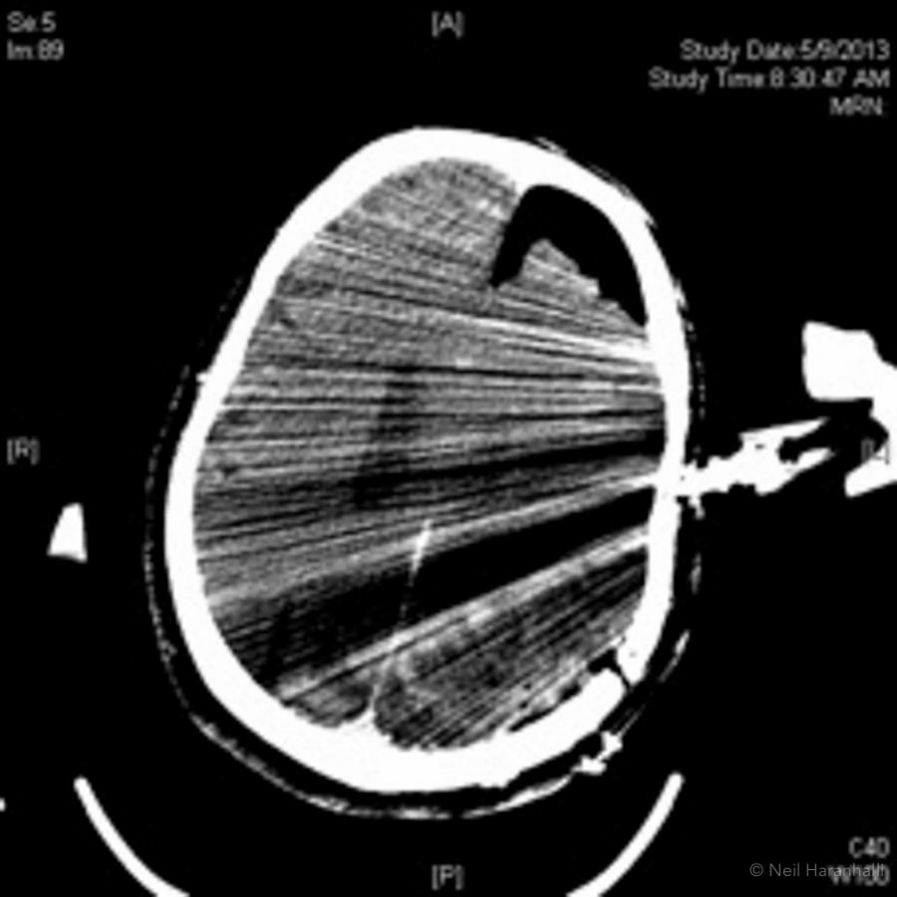
Intraoperative image demonstrating metal streak artifact Note the dark streak in the line of the right pin and the adjacent white streaks. Compared with Fig.1, virtually the entire field of view is obscured. The dark streaks are more prominent compared with Fig.1 due to the lateral parietal bone enhancing the metal streak artifact

During surgery, a radiolucent Doro skull clamp was used (such as used in Figure 1 C) and a BodyTom scanner was done to assess the completeness of neoplasm removal. However, artifacts originating from the skull clamp used made such an evaluation impossible. As can be inferred from Fig. 2, which is similar to Fig. 1, there is a dark streak that comes from the spring on the one pinned arm. Moreover, the entire field of view is obscured by alternating black and white lines which are an expected difference [6, 8-11] when cone beam CT is used compared with conventional CT (Fig. 1). Another possible explanation could be patient motion, which is known to cause this type of artifact [2]. For the images rendered with conventional CT using a phantom, neither problems involving cone beam X-ray nor patient motion were present. Regardless of the cause, the image quality degradation was so severe that the medical utility was highly diminished.

## Discussion

Procedures and radiology have coalesced and evolved for a long time, which is demonstrated, inter alia, by the number of aneurysms that can now be treated endovascularly under the guidance of digital subtraction angiography. For hybrid operating rooms, several portable CT scanners have been developed [12]. Nevertheless, intraoperative imaging might still be in its infancy. With a real-time view of the nervous system and surrounding tissue during surgery, the objective of intraoperative imaging is that neurosurgeons can perform surgery with a higher degree of safety and accuracy. Real-time imaging can be used to minimize the risk of surgical complications and to detect residual tumors [13]. For both low- and high-grade glioma patients, the usefulness of intraoperative MRI was recently demonstrated to increase the amount of resection [14]. However, the full benefit of intraoperative imaging for neurosurgery, especially for imaging with CT, may not be fully evaluated. 

Here, we addressed a specific problem with current skull clamps and pins, i.e. they are not fully radiolucent. We observed that the major source of artifacts is the spring in the one pinned arm. The spring serves to provide a margin of error to the pressure delivered to the skull by tightening of the screw. The most obvious solution would be to remove the spring and replace this protective feature with another protective mechanism. An excellent alternative would be, instead of having a torque screw, to put this protective mechanism outside the frame in the form of a torque screwdriver. Torque screwdrivers are screwdrivers with components that guarantee to tighten to a prespecified torque, ensuring tightening that is sufficient, but not excessive. An insufficiently tightened pin is a surgical problem for obvious reasons, but excessive tightening can also be problematic. Wounds caused by pin-type head holder have been reported to be the source of air entry [15], particularly in the sitting position [16-17], and for epidural hematomas [18-19] and skull fracture [20]. In industry, torque screwdrivers are widely used as part of quality assurance and there is no reason why they cannot be employed in the medical field. Variable clean room torque screwdrivers that can exert torques between 0.02 and 406 Nm exist [21]. The standard Mayfield Torque Screw has a torque of 108.5 Nm and a reduced load 24.5 Nm torque screw exists for procedures in which lower pressure and less pin penetration is desired (Integra specifies the torque in lb, which is not a torque unit. We assumed lb./ft). A skull clamp must resist relatively high forces. A recent study measured the force exerted on a Mayfield head clamp using strain gages during brain surgery, a force range with a median of 5.5 N and a maximum of 151.87 N was measured [22]. There could be an additional advantage of having a variable preset torque screwdriver so that the torque could be adjusted for factors such as age and osteoporosis.

An alternative approach to the problem would be to reduce the metal artifacts with software [1-2, 6-8]. There are various algorithms for this purpose; however, other algorithms must be used for cone beam CT. In the clinical setting, such algorithms have been limited by computer speed [6]. Software for metal streak artifact reduction in cone-beam CT has been recently released and has shown promising in-vitro results. [11]. However, removing the remaining metal in the skull clamp is a more direct solution that is compatible with currently used imaging systems. A third option, which is also less satisfactory, is constructing a spring using a metal with a lower atomic number such as titanium.

## Conclusions

While skull clamps have been used for decades as integral parts of the neurosurgical procedure, the growing use of intra-operative imaging necessitates an improvement of this established equipment. This technical report highlights a few of the key problems with the current devices available in the industry and suggests possible solutions to alleviate the problems faced by surgeons. We believe that the issues discussed in this article are relatively easy to solve and look forward to improved skull clamps in the future that will facilitate improved surgical procedures.

## References

[1] Barrett JF, Keat N (2004). Artifacts in CT: recognition and avoidance. Radiographics.

[2] Boas FE, Fleischmann D (2012). CT artifacts: causes and reduction techniques. Imaging Med.

[3] De Man B (1999). Metal streak artifacts in X-ray computed tomography: a simulation study. IEEE Trans Nucl Sci.

[4] Gossman MS, Graves-Calhoun AR, Wilkinson JD (2009). Establishing radiation therapy treatment planning effects involving implantable pacemakers and implantable cardioverter-defibrillators. J Appl Clin Med Phys.

[5] Farre-Pages N (2011). Relation between bone density and primary implant stability. Med Oral Patol Oral Cir Bucal.

[6] Alexandr Malusek (2008). Calculation of scatter in cone beam CT: steps towards a virtual tomograph. Institutionen for medicin och halsa.

[7] Wang Q (2013). A novel metal artifact reducing method for cone-beam CT based on three approximately orthogonal projections. Phys Med Biol.

[8] Zhang Y (2007). Reducing metal artifacts in cone-beam CT images by preprocessing projection data. Int J Radiat Oncol Biol Phys.

[9] Day Day, J.L J.L (1992). Patent US5276927 A - radiolucent head support. U.S.P. 5276927 Ohio Mechanical Instruments Co..

[10] Mizuho Mizuho (2013). Carbon fiber head frame. http://www.mizuho.com/wp-content/uploads/2014/09/Radiolucent-Head-Frame-Brochure.pdf.

[11] Bechar BB (2012). Metal artefact reduction with cone beam CT: an in vitro study. Br J Radiol.

[12] Rumboldt Z, Huda W, All JW (2009). Review of portable CT with assessment of a dedicated head CT scanner. AJNR Am J Neuroradiol.

[13] Gumprecht H, Lumenta CB (2003). Intraoperative imaging using a mobile computed tomography scanner. Minim Invasive Neurosurg.

[14] Sherman JH (2011). Neurosurgery for brain tumors: update on recent technical advances. Curr Neurol Neurosci Rep.

[15] Pang D (1982). Air embolism associated with wounds from a pin-type head-holder. Case report. J Neurosurg.

[16] Cabezudo JM (1981). Air embolism from wounds from a pin-type head-holder as a complication of posterior fossa surgery in the sitting position. Case report. J Neurosurg.

[17] Wilkins RH, Albin MS (1977). An unusual entrance site of venous air embolism during operations in the sitting position. Surg Neurol.

[18] Baerts WD (1984). Complications of the Mayfield skull clamp. Anesthesiology.

[19] Lee M, Lin EL (2010). The use of the three-pronged Mayfield head clamp resulting in an intracranial epidural hematoma in an adult patient. Eur Spine J.

[20] Matouk CC (2012). Skull fracture secondary to application of a Mayfield skull clamp in an adult patient: case report and review of the literature. Clin Neurol Neurosurg.

[21] (2012). Clean room preset torque limiting screwdriver. Hand torque tool catalog: Mountz MC14 catalog.

[22] De Lorenzo D (2013). Intraoperative forces and moments analysis on patient head clamp during awake brain surgery. Med Biol Eng Comput.

